# Genome-Scale Model Reveals Metabolic Basis of Biomass Partitioning in a Model Diatom

**DOI:** 10.1371/journal.pone.0155038

**Published:** 2016-05-06

**Authors:** Jennifer Levering, Jared Broddrick, Christopher L. Dupont, Graham Peers, Karen Beeri, Joshua Mayers, Alessandra A. Gallina, Andrew E. Allen, Bernhard O. Palsson, Karsten Zengler

**Affiliations:** 1 Department of Bioengineering, University of California San Diego, La Jolla, California, United States of America; 2 Division of Biological Sciences, University of California San Diego, La Jolla, California, United States of America; 3 J. Craig Venter Institute, La Jolla, California, United States of America; 4 Department of Biology, Colorado State University, Fort Collins, Colorado, United States of America; 5 Division of Industrial Biotechnology, Department of Biology and Biotechnology, Chalmers University of Technology, Gothenburg, Sweden; 6 Integrative Oceanography Division, Scripps Institute of Oceanography, University of California San Diego, La Jolla, California, United States of America; Stazione Zoologica Anton Dohrn, Naples, ITALY

## Abstract

Diatoms are eukaryotic microalgae that contain genes from various sources, including bacteria and the secondary endosymbiotic host. Due to this unique combination of genes, diatoms are taxonomically and functionally distinct from other algae and vascular plants and confer novel metabolic capabilities. Based on the genome annotation, we performed a genome-scale metabolic network reconstruction for the marine diatom *Phaeodactylum tricornutum*. Due to their endosymbiotic origin, diatoms possess a complex chloroplast structure which complicates the prediction of subcellular protein localization. Based on previous work we implemented a pipeline that exploits a series of bioinformatics tools to predict protein localization. The manually curated reconstructed metabolic network *i*LB1027_lipid accounts for 1,027 genes associated with 4,456 reactions and 2,172 metabolites distributed across six compartments. To constrain the genome-scale model, we determined the organism specific biomass composition in terms of lipids, carbohydrates, and proteins using Fourier transform infrared spectrometry. Our simulations indicate the presence of a yet unknown glutamine-ornithine shunt that could be used to transfer reducing equivalents generated by photosynthesis to the mitochondria. The model reflects the known biochemical composition of *P*. *tricornutum* in defined culture conditions and enables metabolic engineering strategies to improve the use of *P*. *tricornutum* for biotechnological applications.

## Introduction

Diatoms are unicellular photosynthetic eukaryotes ubiquitous in marine and freshwater habitats and are responsible for about 20% of the photosynthetic carbon fixation on Earth [1]. Diatoms are evolutionary evolved from secondary endosymbiosis and harbor many genes of bacterial origin [2] which is predicted to give these microalgae a wide range of metabolic functions that are distinct from plants, green algae, and red algae [3]. Some of these distinct functions include the formation of silica nanostructures [4], the incorporation of an assimilatory urea cycle [5], and the breakdown of fatty acids in mitochondria and peroxisomes [6]. Diatoms also produce high intracellular concentrations of ω-3 fatty acids and other valuable compounds of biotechnological interest [7].

The marine diatom *Phaeodactylum tricornutum* is an emerging model diatom because of its relatively small genome (27.4 megabases) [2], ease of cultivation, and amenability to genetic engineering. Indeed, genetic systems in *P*. *tricornutum* may be the most advanced in microalgae, with the recently developed ability to assemble whole chromosomes in yeast [8], knock-out genes using TALEN technology [9,10], and introduce stable nucleus-localized episomes the size of small chromosomes via conjugation [11]. Previously developed technologies include transgenic gene overexpression [12] and gene expression knockdown using RNA interference or antisense transcript interference [13]. The development of these genetic engineering systems means that computationally directed experimental manipulations of the diatom genome are not only possible, but necessary.

One promising strategy that investigates the yet unexplored metabolic capabilities of distinct organisms such as *P*. *tricornutum* is the metabolic network reconstruction, which enables computational analysis of systems-level responses. Genome-scale metabolic network reconstructions are derived from the annotated genome and contain information about all known metabolic reactions in an organism including the stoichiometry, subcellular localization, and the gene products by which they are catalyzed. The reconstruction process itself is laborious and iteratively and described, for example, in detail in [14]. The reconstructed network can be transformed into a genome-scale model of metabolism that can be used to predict metabolic phenotypes which are represented by flux distributions and have proven to be useful tools, for example, in the analysis of biological network properties, model-driven discovery, metabolic engineering and strain design [15,16].

Here, we report the reconstruction of a detailed and compartmentalized genome-scale metabolic model for *P*. *tricornutum* which provides a comprehensive insight into yet unexplored metabolic capabilities. We constrained the model with organism-specific biomass equations generated by Fourier transform infrared spectroscopy. The model predicts the presence of a surprising chloroplast glutamine-ornithine shunt that transfers reducing equivalents generated by photosynthesis to the mitochondria. Our findings demonstrate the utility of whole genome metabolic reconstructions to uncover unexpected biochemistries and to provide an important *in silico* template for directing future metabolic engineering efforts.

## Materials and Methods

### Functional genome annotation

The genome annotation of *Phaeodactylum tricornutum* was obtained from JGI (http://genome.jgi-psf.org/Phatr2/Phatr2.home.html). We used the “finished chromosomes” (Phatr2) and “unmapped sequence” (Phatr2_bd) protein sequences to generate the draft reconstruction. While working on the reconstruction, an updated genome annotation, Phatr3, with refined gene models and improved functional annotation became available and exploited as well. Using a Phatr2 to Phatr3 gene ID mapping table provided by the JCVI, the Phatr2 gene IDs in the reconstruction were replaced by their corresponding Phatr3 IDs. Phatr3 is available at Ensembl Protistis (http://protists.ensembl.org/Phaeodactylum_tricornutum/Info/Index).

The *P*. *tricornutum* genome annotation contains many putative enzymes with unknown function. To facilitate the manual curation of our draft reconstruction we used protein BLAST (with default settings) to reannotate the predicted proteins. Using the BLAST command line tool we created a local BLAST database containing all reviewed UniProtKB/SwissProt sequences having protein evidence at the protein or transcript level [17]. Using an in-house IPython Notebook script we performed a bidirectional best hits analysis between the predicted *P*. *tricornutum* proteins and the created UniProt BLAST database.

### Subcellular localization prediction pipeline

To predict a subcellular localization for each protein we used a refined version of a previously developed pipeline. We used the updated Phatr3 protein sequences as input for TMHMM 2.0 [18], Mitoprot II 1.101 [19], SignalP 3.0 [20], SignalP 4.0 [21], TargetP 1.1 [22] and HECTAR [23]. All programs were run using default settings. The resulting files were parsed using in-house bash scripts and integrated into a single pipeline which was implemented using IPython Notebook and Pandas.

We extended the pipeline by i) removing nuclear targeted proteins using predictNLS [24], ii) screening for chloroplast periplasm targeting prior to evaluating for the occurrence of an endoplasmic reticulum (ER) retention signal, iii) searching for the peroxisome signal in the very last three C-terminal amino acids, and iv) allowing concomitant localization of proteins to mitochondria and peroxisome. More details on the prediction pipeline are given in Section A in S1 File.

### Organism-specific biomass composition

Based on the experimental approach of Mayers *et al*. [25] and [26–29], we determined the biomass composition in terms of lipids, fatty acid methyl ester (FAME), carbohydrates, and proteins using traditional biochemical methods while also examining the Fourier transform infrared (FTIR) spectrometry profiles of lyophilized and homogenized cell pellets. The biochemical measurements were used in a calibration against their corresponding FTIR peaks, with the methods described in detail in Section B in S1 File. These dual measurements were then used to develop linear models with spectra peak height and *P*. *tricornutum* biochemical composition (essentially linear correlation curves) as done by Mayers *et al*. [25].

By conducting these measurements over a growth curve including samples from nitrogen replete during exponential growth phase to nitrogen starved during stationary phase, we were able to achieve large changes in the cellular contents for all of these cellular components in smooth gradients (Tables A-C in S2 File). This, in turn, allowed us to develop models correlating FTIR spectra peak heights to cellular composition, thereby facilitating higher-throughput determinations of cellular biomass composition (Fig A in S1 File, Section B in S1 File). Based on our experimental data (Tables A-C in S2 File) and previous work [30–38] the biomass equation was set up as described in Section C in S1 File and Tables D-L in S2 File. The experimental workflow is depicted in Fig B in S1 File.

### Network reconstruction and modeling simulations

Since the general reconstruction process has been described in detail elsewhere [14] we only provide procedural details specific to this work. To build a draft reconstruction, three reference models from related photosynthetic organisms were exploited; one network for *Chlamydomonas reinhardtii* (*i*RC1080 [39]), and two genome-scale models for *Synechocystis* sp. PCC6803 (*i*JN678 [40] and Knoop [41]). Before reconciling the reference networks, we removed the compartmental pH from *i*RC1080 and implemented all metabolites at a pH of 7.1. This step facilitated the metabolite reconciliation of the reference networks based on metabolite formulas. We also made sure that none of the reference networks contained nested gene reaction associations and expanded each reaction into several reactions, each under the control of only one enzyme. We reconciled the reference network’s metabolite and reaction abbreviations using the modelBorgifier Toolbox [42]. We used *i*RC1080 as the template model and subsequently compared *i*JN678 and Knoop to the template model.

Starting from the *P*. *tricornutum* genome annotation Phatr2 (Phatr3 was not yet available) and the reconciled reference networks we obtained a draft reconstruction based on homology using the RAVEN Toolbox [43]. Before proceeding with the manual curation, we i) checked reactions associated to genes from *Chlamydomonas* or *Synecchocystis* for which no homologs in *P*. *tricornutum* were found and verified whether these reactions are present in *P*. *tricornutum* or not, ii) merged expanded reactions, iii) removed compartments not relevant for *P*. *tricornutum*, e.g., the eyespot, iv) removed duplicated metabolites and reactions which were introduced due to incorrectly reconciled information, and v) edited annotations.

We manually curated the draft reconstruction pathway-by-pathway and verified the given information and added any missing information using the COBRA Toolbox [44]. Besides the genome annotation, several other resources were exploited, such as primary literature, DiatomCyc [45], KEGG [46], and UniProt [17]. Information regarding transport proteins was obtained from TransportDB [47] and TCDB [48].

For each reaction in the *P*. *tricornutum* reconstruction, the involved metabolites were characterized according to their chemical formula and charge determined at a pH of 7.3 using MarvinSketch (ChemAxon, http://www.chemaxon.com/products/marvin/marvinsketch). The pH was presumed to be constant across all compartments due to missing information for *P*. *tricornutum*. All reactions were elementally and charge balanced. Reaction reversibility was chosen based on published reconstructions such as *i*RC1080 or according to databases such as BIGG [49] or SimPheny^™^ (Genomatica Inc., San Diego, CA).

Protein subcellular localization was assigned based on the prediction pipeline and indirect physiological evidence. If available, protein localization data from experiments with transgenic diatoms expressing protein-fluorescent protein fusions was exploited. Gene-reaction associations were identified from the literature, genome annotation, or genome sequence using BLAST and formulated as Boolean logic statements. Based on the biological evidence found we assigned a confidence score to each reaction reflecting the available information and evidence for its inclusion [14]. Here, the confidence scores range from 1 to 5, with 1 being low confidence and 5 representing very high confidence (see Table N in S2 File).

Since naming might be ambiguous, different identifiers were used to annotate the reactions and metabolites. Reactions were annotated with EC numbers and KEGG reaction identifiers, metabolites were annotated with KEGG compound, ChEBI, and InChI identifiers.

Each reaction was associated with at least one subsystem similar to the subsystem naming convention used in the KEGG database [46]. Exchange reactions were added to enable uptake and secretion of extracellular metabolites for the purpose of simulations.

Quality control was performed during the reconstruction process. We ensured that ATP could not be produced without inputs. This was tested according to established standards [14] by optimizing the flux through the ATP maintenance reaction while closing CO_2_ and photon uptake. To validate that NAD(P) production did not occur without nutrient uptake we introduced an artificial reaction NAD(P)H → NAD(P) + H and again closed CO_2_ and photon uptake. If we found ATP production in the absence of nutrients, we identified all reactions contributing to the flux and produced a metabolic map using Escher [50] in order to distinguish between type III pathways and reactions involved in ATP production. The reactions involved in ATP production were reviewed manually.

### Modeling simulations

Mathematically, the reconstruction is represented by the stoichiometric matrix **S** (m x n) where m is the number of metabolites and n is the number of reactions. The entries in the stoichiometric matrix, s_ij_, represent the stoichiometric coefficients for the participation of the i^th^ metabolite in the j^th^ reaction. A negative value indicates consumption of metabolite i in reaction j whereas s_ij_ > 0 represents production of metabolite i. Flux balance analysis (FBA, [51]) was used to solve the linear programming (LP) problem under steady-state criteria represented by the equation **S**∙**v** = 0 where **v** is a vector of reaction fluxes.

To constrain the space of possible solutions, the biomass objective function accounting for the ratios of biomass components (e.g., lipids) and biomass precursors (e.g., amino acids) as well as energetic requirements to produce 1 g of biomass, is optimized for.

One challenge of metabolic models for phototrophic organisms is applying constraints such as nutrient uptake, photon absorption and product secretion to simulate phenotypic behavior. Phototrophic metabolism was simulated by constraining the maximal nitrogen and carbon uptake according to our experimental data. The nitrogen uptake was set based on cellular nitrogen levels determined by elemental analysis assuming that excreted metabolites were negligible during exponential growth (Table L in S2 File). Carbon uptake was enforced by setting the lower bound of the CO_2_ exchange reaction to the experimentally determined total organic carbon (Table L in S2 File).

LP calculations were performed using the Gurobi Optimizer Version 6.0.4 (Gurobi Optimization Inc., Houston, Texas) solver in MATLAB (The MathWorks Inc., Natick, MA) with the COBRA Toolbox [44].

### Carbon partitioning

Dark period culture measurements were taken after the cells completed division; evidenced by consistency in the cell counts between dark and light period samples. Therefore, we hypothesized all biomass increases during the light period resulted from assimilation of extracellular nutrients. Elemental analysis indicated the culture fixed 1.57 mM C and assimilated 0.535 mM N during the light phase on culture day 5 (samples 8 and 9, see Table L in S2 File). These values were used as the upper bounds for CO_2_ and NO_3_ uptake. The ATP maintenance reactions were set to a range of 0–1 mM based on experimental results indicating negligible maintenance requirements [52].

However, unlike the traditional biomass function where the stoichiometry is pre-determined, dynamic allocation of fixed carbon was possible through the implementation of demand reactions for a β-1,3-glucose molecule representing the diatom storage glycan, chrysolaminarin, and TAG(16:1Δ9/16:1Δ9/16:0), the most abundant storage TAG observed during nutrient replete growth in *P*. *tricornutum* [37]. Additional demand reactions included ammonia (nh4_h) and DMSP (dmsp_c). Photon uptake was varied from 0 to 50 mM photon to determine the super-saturating photon uptake value of 22 mM at which the simulations were performed. The objective function was set to maximize CO_2_ uptake with a secondary objective of minimizing the Manhattan norm of the flux vector representing the cell’s strategy to minimize the sum of flux values [53]. To simulate energetic coupling between the plastid and mitochondria, the model was constrained with the inequality *v*_NADHOR_m_−C∙*v*_PSI_u_ ≥ 0 where *v*_NADHOR_m_ is the flux through the oxidative phosphorylation complex I, *v*_PSI_u_ is the flux through photosystem I (a proxy for total electron flow), and C > 0 represents the minimal fraction of total photosynthetically fixed electrons that have to be directed to the mitochondria.

## Results and Discussion

### Metabolic network reconstruction

**Fig 1:**
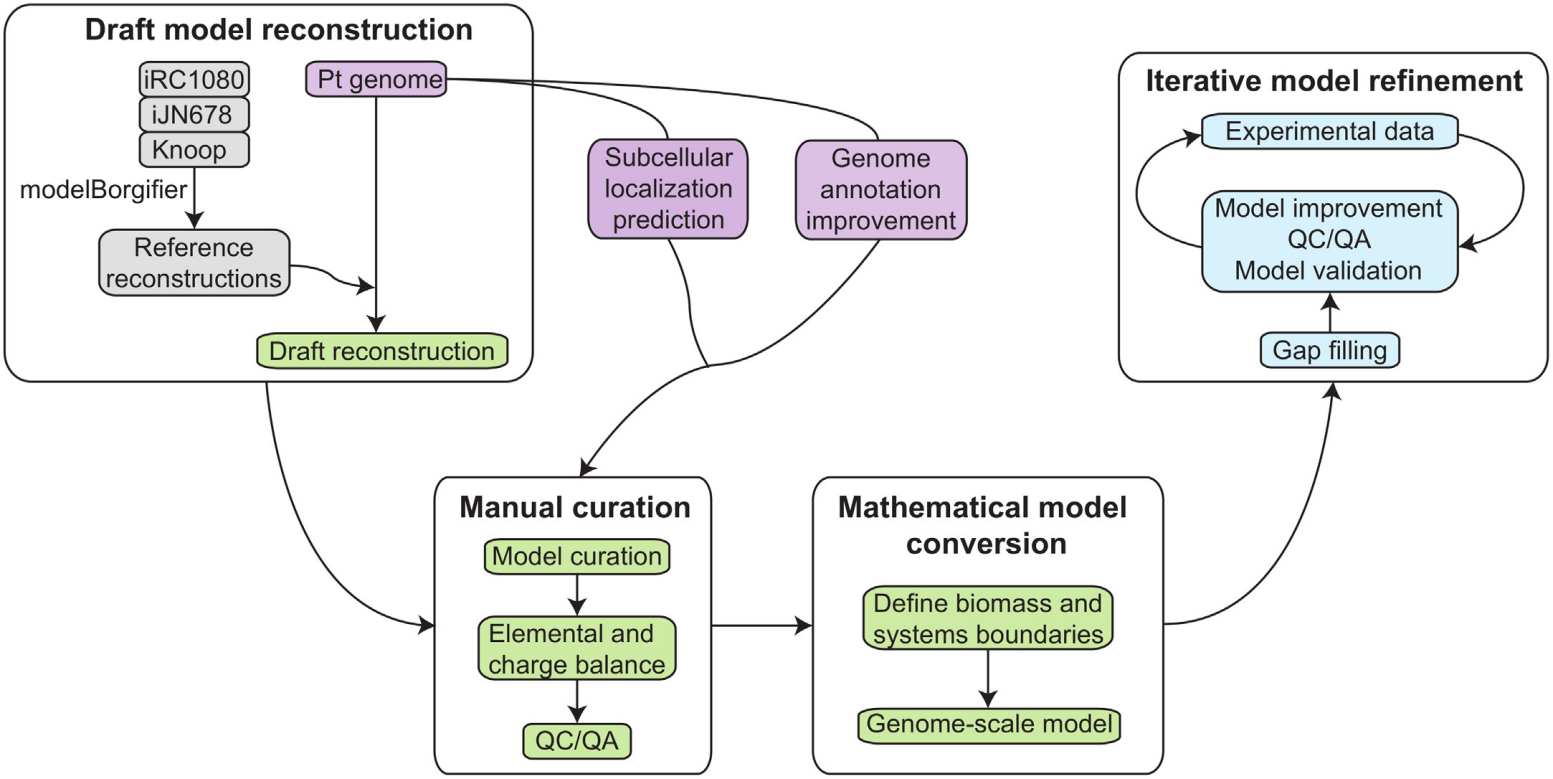
Metabolic network reconstruction workflow. In step one we obtained a draft reconstruction based on *P*. *tricornutum*’s genome annotation and reference reconstructions. This draft reconstruction was manually curated using several resources such as an improved genome annotation, subcellular localization predictions and external databases. All reactions were elementally and charge balanced, QC/QA was performed and a biomass objective function was defined before transforming the reconstruction into a computational model. In an iterative process, the *in silico* predictions are compared with experimental observations to validate and improve the metabolic model.

Genome-scale network reconstructions are biochemically, genetically and genomically structured knowledge-bases which provide a framework to analyze and predict genotype-phenotype relationships. The reconstruction process is divided into four main steps [14] and summarized in Fig 1.

First, we generated a draft reconstruction based on the *P*. *tricornutum* genome annotation and protein homology to template organisms having reconstructions [39–41]. Diatoms are taxonomically and functionally distinct from other algae and vascular plants; in fact, many nuclear genomic contents are more closely related to metazoans, demonstrating the diversity of diatom metabolism [2]. Although the diversity complicated the generation of a homology-based draft reconstruction, it also makes diatoms, such as the model organism *P*. *tricornutum*, attractive candidates for the analysis of cellular processes at a systems level, as they add to the biochemical diversity of microbes in a biotechnology setting, thereby increasing available production systems. Second, the draft reconstruction was manually curated and refined using additional resources such as the genome annotation, subcellular localization predictions and external databases (see Materials and Methods). Once the manual curation was completed, the reconstruction was converted into a mathematical model in the third step. We added the biomass objective function and defined system boundaries (i.e., carbon and nitrogen uptake) according to experimental results (see Materials and Methods). Qualitative tests were performed during the manual curation and the final step of model refinement and analysis. We verified that all biomass components and vitamins for which *P*. *tricornutum* is autotrophic could be produced under realistic growth conditions. Blocked pathways could be resolved with the addition of one or two reactions; in most cases transport reactions between intracellular compartments were missing. Furthermore, we ensured that ATP could not be produced without inputs. We also performed several *in silico* tests to assess the consistency of our model and verify that known physiological behaviors can be computationally reproduced. Diatoms are able to utilize a variety of nitrogen sources, both inorganic (such as nitrate and ammonium [54]) and organic (e.g. amino acids or urea [55]). Therefore, we examined the ability of the model to simulate biomass production on different nitrogen sources. Biomass was not produced in the presence of histidine, tryptophan, cysteine, or methionine as sole nitrogen sources in our initial *in silico* model, which contradicted literature results [55]. Histidine catabolism is not well understood in diatoms or plants and was not incorporated in the model at first. Since we could not identify genes that are involved in histidine catabolism in *P*. *tricornutum*, we added histidine catabolism as one lumped, low confidence reaction degrading histidine and water into ammonium, formamide and glutamate. Formamide is split into formate and ammonium with formate accumulating during histidine catabolism *in silico*; a demand reaction was added to allow the accumulated formate to leave the system. Biomass production for growth on methionine or cysteine as sole nitrogen sources was achieved by adding a demand reaction for dimethylsulphoniopropionate (DMSP). DMSP levels are known to increase with light intensity or nitrogen starvation but its metabolism is not well understood in diatoms and while the biosynthetic pathway is currently unknown [56], a sensible starting point would be an amino acid with an already reduced sulfur atom. Indole accumulation prohibited growth on tryptophan as nitrogen source. To account for the unknown indole degradation, a demand reaction was added. With these changes, the model could simulate biomass production using the different nitrogen sources tested.

Leveraging a genome-scale model in the exploration and contextualization of lipid metabolism requires an accurate representation of the metabolic pathways and intermediate metabolites. To this end, a lipid module was developed (*i*LB1027_lipid, see S3 File) that encompasses the full range of lipid metabolites and metabolic reactions. This module allows incorporation of experimental fatty acid and lipid class characterization to be reflected in the biomass composition. Incorporation of experimental FAME data was possible via a linear optimization based data fitting algorithm (see Materials and Methods). After fitting the model to the data, the deviation from the experimental values to the model was 350 times lower in the lipid module compared to the core model. This result demonstrates the utility of the lipid module when investigating fatty acid and lipid metabolism in *P*. *tricornutum*.

**Fig 2:**
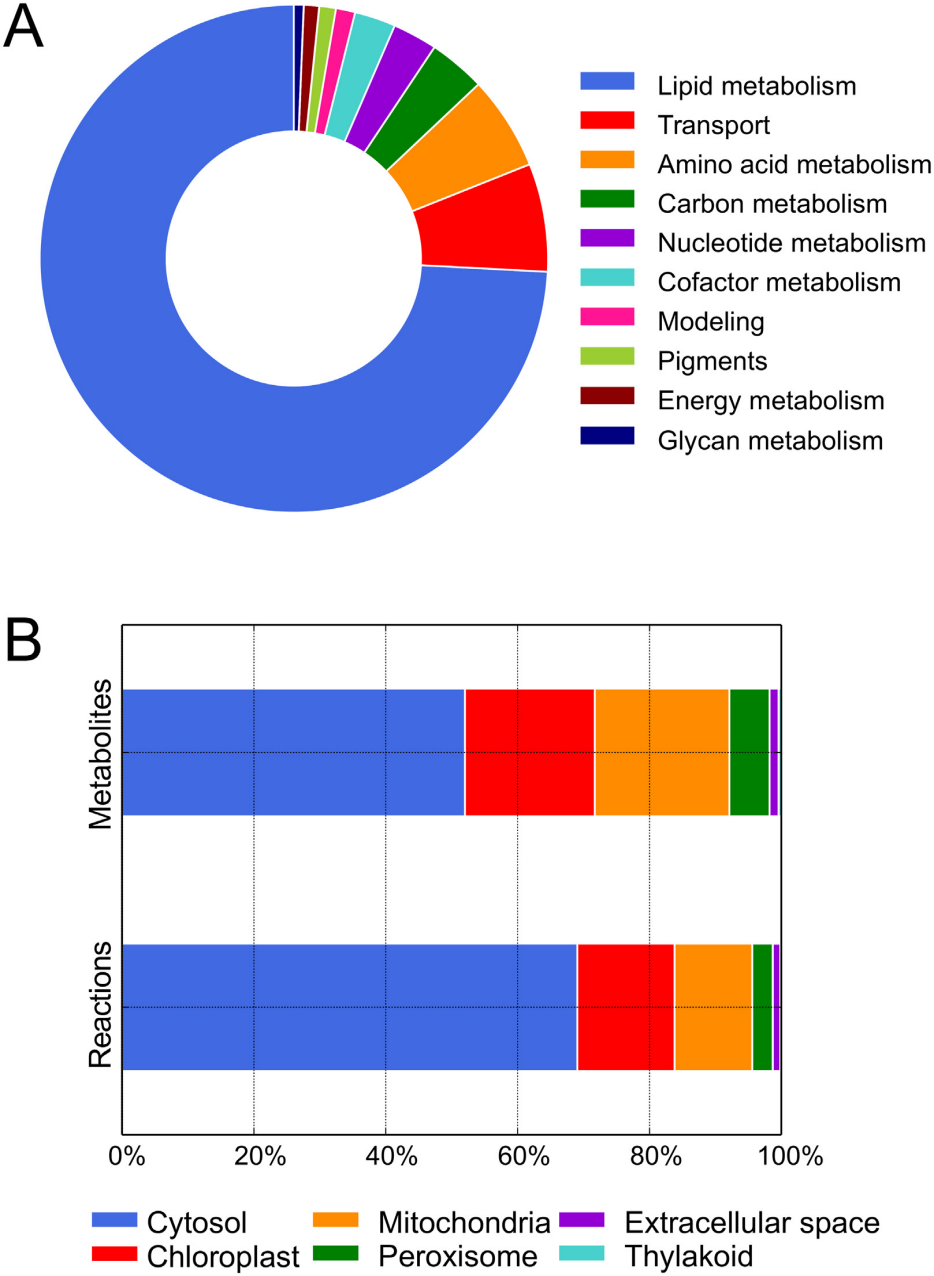
Reconstruction characteristics *i*LB1027_lipid. (A) Reactions per subsystem. Most reactions are involved in lipid metabolism. Our FTIR measurements underline the fact that the lipids make up the highest fraction of biomass. Due to the presence of multiple compartments and the fact that many pathways are split among compartments, many reactions are attributed to intracellular transport. The modeling subsystem contains ATP maintenance, biomass, demand, sink, and exchange reactions. (B) Percent reactions and metabolites per compartment. Most reactions and metabolites are present in the cytosol, followed by chloroplast and mitochondria in the case of reactions and mitochondria and chloroplast for metabolites. Peroxisome, extracellular space, and thylakoid contain less than 5% and 8% of all reactions and metabolites in the reconstruction, respectively.

The curated genome-scale metabolic network for *P*. *tricornutum* including the lipid module, *i*LB1027_lipid, accounts for 1,027 genes associated with 4,456 reactions and 2,172 metabolites distributed across six compartments (Tables M-O in S2 and S3 Files). Compared to the draft reconstruction, the number of genes (446 genes) was more than doubled during the manual curation phase. All reactions are associated with at least one of 90 subsystems which can be categorized into ten groups, e.g., carbon or lipid metabolism (Fig 2). Additionally, a core model with substantially reduced lipid metabolism (*i*LB1025) was constructed. The reduced lipid metabolism subsystem accounts for 1,029 reactions compared to 3,325 reactions involved in lipid metabolism in *i*LB1027_lipid. The core model yields comparable flux distributions and is suitable, for example, if detailed data on the lipid composition under the simulated condition are missing.

### Prediction of enzyme subcellular localization

**Fig 3:**
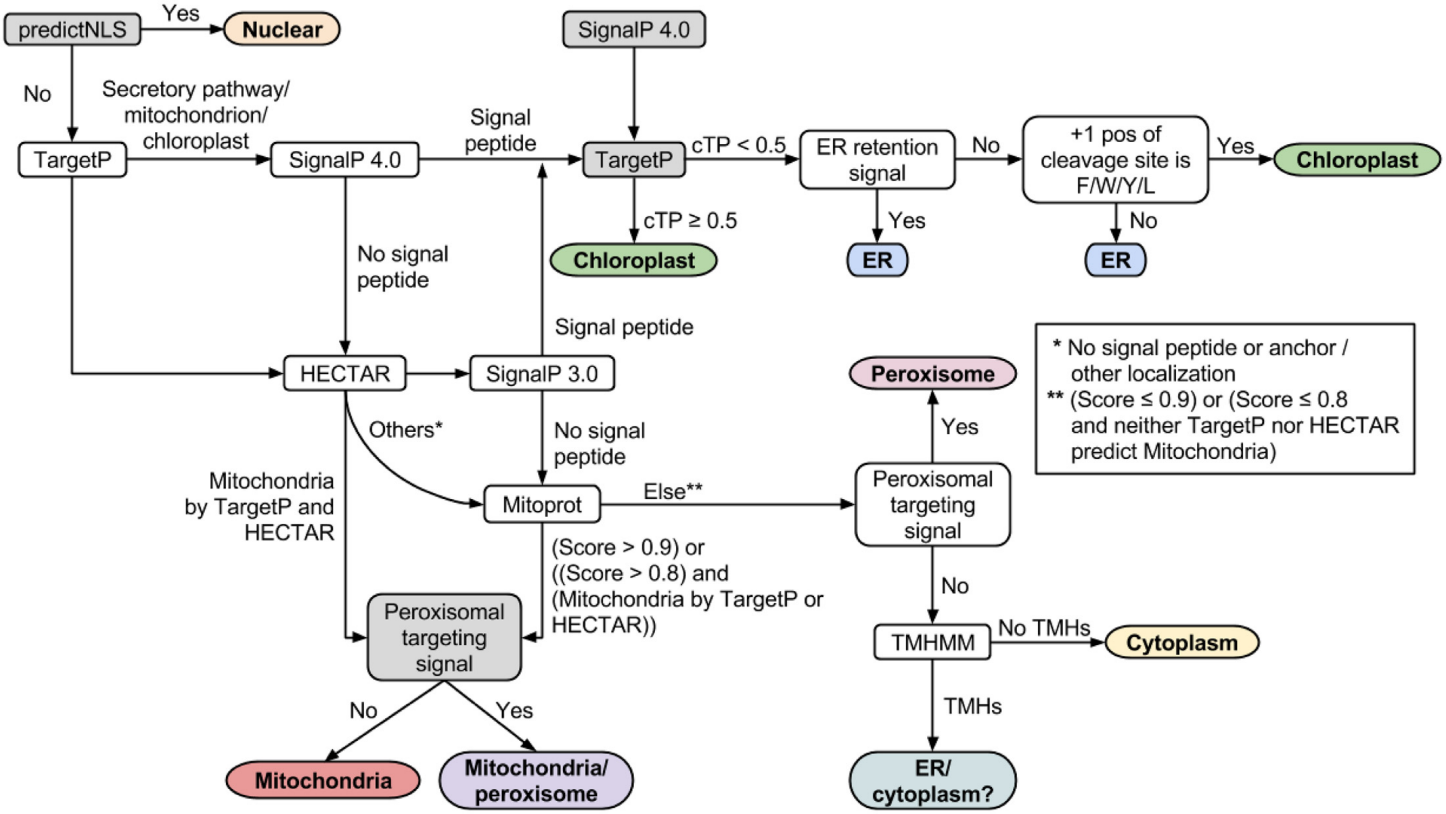
Subcellular localization prediction pipeline. Schematic representation of the implemented subcellular localization prediction pipeline for *Phaeodactylum tricornutum* adapted from previous work [57]. Subcellular compartments are given in ellipses and bioinformatics programs are displayed in rectangles. Our added steps are highlighted in gray. The ER retention signal is (K/D)-(D/E)-E-L in the protein C-terminal region. A protein is categorized as peroxisomal if the signal (S/A/C)-(K/R/H)-(L/M) or S-S-L is found in the C-terminal region.

One challenging aspect of eukaryotic reconstructions is the subcellular localization prediction of proteins. Due to their endosymbiotic origin, photosynthetic heterokonts including diatoms possess chloroplasts that are surrounded by four membranes. This complex structure concurs with distinct plastid targeting signals in diatoms that restrict the use of available subcellular prediction tools for other eukaryotes. We enhanced a previously developed pipeline which combined different bioinformatics programs to predict the subcellular localization of proteins in diatoms [57] (see Fig 3, Materials and Methods, and Section A in S1 File).

To evaluate the accuracy of the improved pipeline, we compared our predictions to Sunaga *et al*.’s results and experimentally validated subcellular protein localizations taken from [5,12,58–64]. By using the refined pipeline, 15 out of 19 subcellular localization predictions coincided with experimental data as summarized in Table 1.

**Table 1:** Validation of the *in silico* subcellular localization prediction pipeline.

Protein	ID		Status Phatr3	Experimental	Prediction original pipeline		Prediction improved pipeline		TargetP	MitoProt	Note
	Phatr2	Phatr3		localization	Phatr2	Phatr3	Phatr2	Phatr3			
Fructose-bisphosphate aldolase	Phatr_825_bd	304098	Kept	Chloroplast [58]	Chloroplast	Chloroplast	Chloroplast	Chloroplast	ER	0.2507	
Glyceraldehyde-3-phosphate dehydrogenase	Phatr_25308	301292	Kept	Mitochondrion [59]	Mitochondrion	Mitochondrion	Mitochondrion	Mitochondrion	Mitochondrion	0.9647	
Glyceraldehyde-3-phosphate dehydrogenase	Phatr_22122	308678	Kept	Chloroplast [59]	Chloroplast	Chloroplast	Chloroplast	Chloroplast	Chloroplast	0.946	
Transaldolase	Phatr_20779	304241	Kept	Chloroplast [58]	Chloroplast	Chloroplast	Chloroplast	Chloroplast	Mitochondrion	0.9717	
Glutamine synthetase	Phatr_22357	310769	Kept	Mitochondrion [12]	Mitochondrion	Mitochondrion	Mitochondrion	Mitochondrion	Mitochondrion	0.9972	
Glutamine synthetase	Phatr_51092	306624	Kept	Chloroplast [12]	Chloroplast	Chloroplast	Chloroplast	Chloroplast	ER	0.4399	
*Carbamoyl phosphate synthase*	*Phatr_24195*	*309585*	*Modified*	*Mitochondrion [5]*	*Cytoplasm*	*Mitochondrion*	*Cytoplasm*	*Mitochondrion*	*Mitochondrion*	*0*.*9588*	*As mentioned by Sunaga et al*., *the Phatr2 gene model is incomplete*. *Usage of Phatr3 gene model yields correct prediction*.
Fructose-1,6-bisphosphatase	Phatr_54279	311663	Kept	Chloroplast [58]	Chloroplast	Chloroplast	Chloroplast	Chloroplast	ER	0.1645	Phatr2 ID 2793 shorter version of 54279.
*Δ12 desaturase*	*Phatr_48423*	*300552*	*Kept*	*Chloroplast [60]*	*ER*	*ER*	*Chloroplast*	*Chloroplast*	*Chloroplast*	*0*.*813*	*Usage of improved pipeline yields correct prediction*. *Note that we could not reproduce Sunaga et al*.*'s result using the original pipeline*.
ATPase δ subunit	Phatr_20657	301964	Modified	Chloroplast [61]	Chloroplast	Chloroplast	Chloroplast	Chloroplast	Chloroplast	No prediction	
PtCA1	Phatr_51305	306874	Kept	Chloroplast [62]	Chloroplast	Chloroplast	Chloroplast	Chloroplast	ER	0.5189	
Triosephosphate translocator	Phatr_24610	308968	Kept	Chloroplast [63]	Chloroplast	Chloroplast	Chloroplast	Chloroplast	ER	0.6211	
*CA-I*	*Phatr_35370*	*303871*	*Kept*	*Chloroplast [64]*	*ER*	*ER*	*Chloroplast*	*Chloroplast*	*Mitochondrion*	*0*.*4648*	*Usage of improved pipeline yields correct prediction*.
**CA-II**	**Phatr_44526**	**311660**	**Modified**	**Chloroplast [64]**	**ER**	**ER**	**ER**	**ER**	**ER**	**No prediction**	**Wrong prediction.**
**CA-III**	**Phatr_55029**	**300968**	**Modified**	**Chloroplast [64]**	**ER**	**No prediction**	**ER**	**No prediction**	**-**	**No prediction**	**For Phatr_55029, the pipeline predicts endoplasmic reticulum instead of chloroplast. The improved gene model 300968 was extended at the N-terminus and does not start with methionine anymore. Mitoprot does not predict localization if the protein does not start with methionine and, therefore, the pipeline does not predict localization for the Phatr3 gene model.**
PtCA2	Phatr_45443	311919	Kept	Chloroplast [62]	Chloroplast	Chloroplast	Chloroplast	Chloroplast	ER	0.4762	
**CA-VI**	**Phatr_54251**	**303635**	**Kept**	**Chloroplast [64]**	**ER**	**ER**	**ER**	**ER**	**ER**	**0.3987**	**Both pipeline versions predict endoplasmic reticulum instead of chloroplast.**
**CA-VII**	**Phatr_42574**	**304857**	**Modified**	**Chloroplast [64]**	**ER**	**ER**	**ER**	**ER**	**ER**	**No prediction**	**Both pipeline versions predict endoplasmic reticulum instead of chloroplast.**
CA-VIII	Phatr_20030	311877	Kept	Mitochondrion [64]	Mitochondrion	Mitochondrion	Mitochondrion	Mitochondrion	Mitochondrion	0.932	Phatr2 ID 35304 shorter version of 20030.

The table compares predictions of protein localizations to experimental data. For all considered proteins, Phatr2 and Phatr3 IDs and the status of the gene model in Phatr3 are given. If the gene models were modified, the pipeline predictions for both gene models are given. We distinguish between two versions of the *in silico* pipeline; original refers to the version as published by Sunaga *et al*. [57] and the improved version is the one presented in this study. Entries for which the improved pipeline or usage of Phatr3 gene models improved the prediction are formatted italic. Discrepancies between prediction and experimental localization are shown in bold. ER: Endoplasmic reticulum.

### Determination and modeling of biomass composition

In order to mathematically solve the genome-scale model using FBA, the observed cellular phenotype is manifested as a biological objective function [51]. This objective function is a metabolic reaction in the model that is maximized or minimized in order to achieve a desired phenotypic state. In order to simulate cellular growth, the macromolecular constituents of the cell are defined as the objective function (see Table L in S2 File). This biomass objective function accounts for all known cellular components and their fractional contributions to the overall cellular biomass, defines the anabolic requirements for cell division, and provides mass balance.

The biomass composition used in heterotrophic genome-scale models is typically fixed based on experimentally derived values at a given culture condition [65]. However, phototrophic organisms have a dynamic biomass composition that changes not only across the diel cycle, but also along the duration of the culture. In *P*. *tricornutum*, biomass changes in the light period is dominated by the generation of carbon storage compounds, while the dark period is dominated by the anabolic processes necessary for cell division [66]. There is also dramatic remodeling of the cellular biomass composition that accompanies nutrient limitation in diatoms [67].

**Fig 4:**
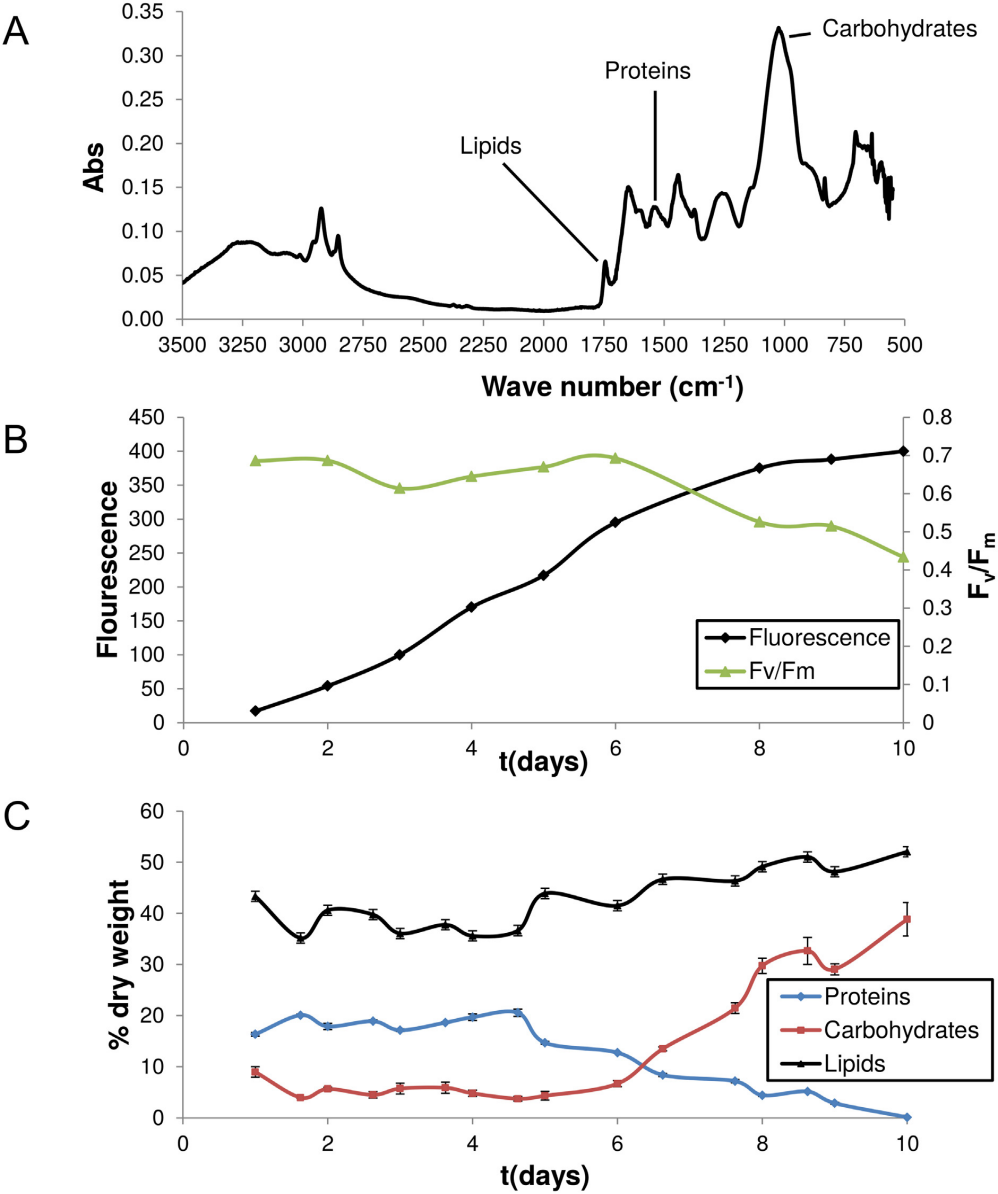
FTIR spectrum and culture data. A typical FTIR spectrum for *Phaeodactylum tricornutum* is shown in (A). Peaks corresponding to lipids, proteins and carbohydrates are highlighted (see Table A in S1 File for specific wavelengths). Panel (B) shows the growth curve and photosynthetic efficiency of the culture used for model calibrations and the biomass objective function. The decline in F_v_/F_m_ indicates the onset of nitrogen starvation (n = 1). Percent dry weight of the cells in terms of carbohydrates, lipids, and proteins according to FTIR spectra and the calibrated linear model (n = 5, error bars represent five independent FTIR scans) is displayed in (C).

High confidence intracellular flux predictions are dependent on the biomass composition being accurately reflected during the simulation. To this end, we determined *P*. *tricornutum*’s biomass composition over a growth curve that resulted in nitrogen deprivation after the high accumulation of biomass (Fig 4). Selected samples of this growth curve were examined using time consuming biochemical methods for determining lipid, carbohydrate, and protein content of the cells. Parallel samples were used to develop linear models relating FTIR peaks to biomass composition (Fig A in S1 File). These calibrated models were then used to determine the biomass composition for all time points. The linear models are most robust when a large gradient for biomass composition values (i.e., percent lipid, protein, and carbohydrate) are achieved, thus our experiment was designed to maximize the changes in content. Nitrogen starvation, low CO_2_, and low light all can contribute to high lipid content and all three scenarios were achieved in our engineered culture experiment, resulting in very high lipid values at the end of the experiment (Fig 4C). The lipid values are elevated relative to previous experiments that examined more realistic bioproduction conditions, but this was planned and resulted in the expected fashion. We were able to achieve large changes in the cellular contents for all of these cellular components in smooth gradients.

Additionally, FAME data at each sample point was incorporated into the biomass composition via a linear optimization based fitting algorithm to ensure changes in fatty acid biosynthesis were taken into consideration during simulations (see Materials and Methods and Section B in S1 File). Interestingly, diatoms store large amounts of nitrogen in the cell in the form of inorganic compounds [30], probably in the vacuole [68]. A demand reaction for NO_3_ was added to account for cellular nitrate that has not yet been assimilated into other biomass components such as proteins but is included in the dry weight measurements. By defining the cellular composition at each sampling point, differences in the metabolic network usage could be analyzed along the duration of the culture.

Commonly, maximizing the biomass equation is selected as an appropriate objective function for the growth phenotype. Since cell division in *P*. *tricornutum* is relegated to the dark period when cells are grown in a light-dark regimen, the common biological objective function of maximizing growth is not applicable to simulations during the light period. Thus, maximizing carbon uptake was selected as the biological objective function that best represents the cellular phenotype during the light period. Mass balance was achieved by allowing fixed carbon to accumulate as either carbohydrates or neutral lipids in accordance with previous observations of *P*. *tricornutum* [66].

### Comparison to other models

Several metabolic models for *P*. *tricornutum* have been constructed to date (Table 2). Kroth and coworkers investigated the localization of enzymes and pathways involved in carbohydrate metabolism [69]. This model served as foundation for the first genome-scale model for *P*. *tricornutum* which was presented in form of a detailed pathway/genome database named DiatomCyc [45]. DiatomCyc comprises a high number of pathways and offers different software tools, e.g. for network analysis, but it lacks subcellular compartments which are important to account for distinct environments required for different metabolic processes. A smaller and compartmentalized version of the DiatomCyc metabolic network was used to compute elementary flux modes and investigate light-dependent changes in *P*. *tricornutum*’s metabolism [70,71]. Here, little information about the reconstruction process is given and reactions and metabolites are poorly annotated. Kim *et al*. developed the most recent genome-scale metabolic network for *P*. *tricornutum* and explored flux distributions for autotrophic, mixotrophic and heterotrophic growth conditions [72]. For all three modes, the same biomass objective function was exploited. The prediction of protein localization was based on MitoProt [19] and TargetP [22]. Reactions are annotated using EC numbers which might be ambiguous and hamper clear identification of reaction mechanism or model comparison based on reaction content. Gene reaction associations are not formulated as Boolean rules making it impossible to distinguish between isozymes, enzyme complexes, or subunits. No information about the performance of quality control or mass and charge balancing is given.

**Table 2:** Characteristics of available models for *Phaeodactylum tricornutum*.

Property	Kroth *et al*. [69]	DiatomCyc [45]	Hunt *et al*. [71]	Kim *et al*. [72]	*i*LB1027_lipid (this study)
**Reactions**	88	1719 metabolic reactions 67 transport reactions	318	849 (not including biomass equation)	4456 (*i*LB1025: 2156)
**Metabolites**	Not available	1173	335	587	2172 (*i*LB1025: 1704)
**Genes**	151	1613	680	607	1027 (*i*LB1025: 1025)
**Compartments**	Cytoplasm, Mitochondria, Chloroplast, Endoplasmic reticulum, Peroxisome	Cytoplasm	Cytoplasm, Mitochondria, Chloroplast, Peroxisome	Cytoplasm, Mitochondria, Chloroplast (stroma and lumen), Peroxisome	Cytoplasm, Mitochondria, Chloroplast (stroma and thylakoid), Peroxisome
**Reconstruction software**	Not available	Pathway Tools	CellNetAnalyzer	MOST	COBRA Toolbox, COBRApy
**Availability**	No mathematical model available	Online access	SBML	SBML	SBML, MAT
**Notes**	Carbohydrate metabolism	Genome-wide model, not compartmentalized	Simplified and compartmentalized version of DiatomCyc; see [70] for simulations	Genome-wide model, GPRs not in Boolean format	Genome-wide model, detailed lipid metabolism

Metabolic model characteristics are compared between four available models for *P*. *tricornutum* and the one presented in this study.

Here, we based our reconstruction effort on the updated and improved genome annotation which yields more precise localization predictions due to refined gene models. Compared to predictions of each bioinformatics tool, the sophisticated protein localization pipeline more often coincides with experimental findings (Table 1). Since diatom metabolism and consequently biomass components strongly vary with growth conditions (Fig 4), we determined *P*. *tricornutum*’s biomass composition over a growth curve that resulted in nitrogen deprivation after the high accumulation of biomass.

**Fig 5:**
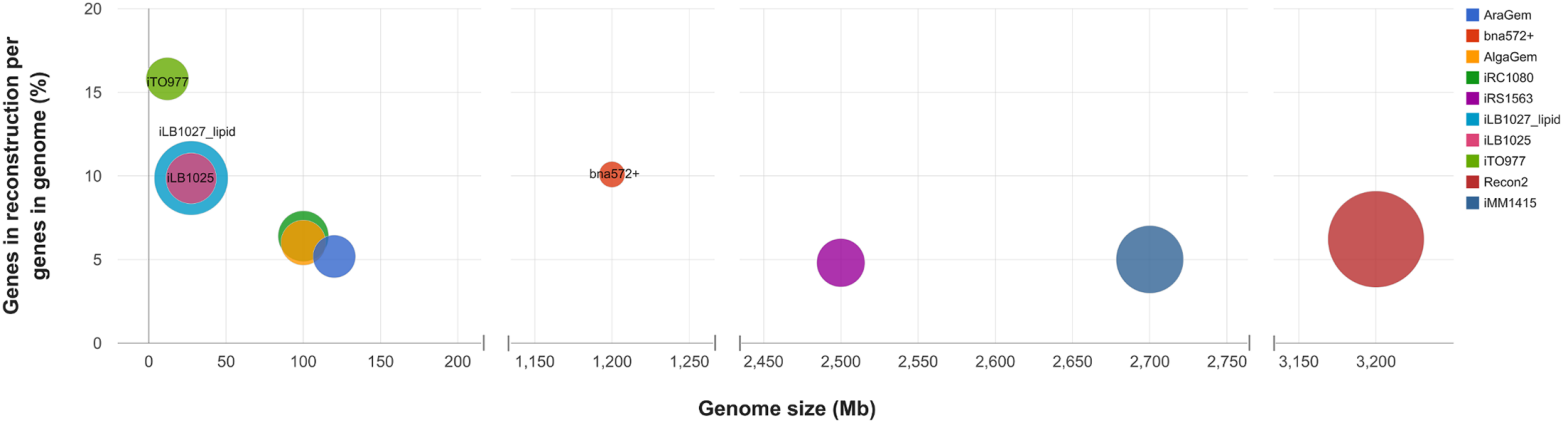
Genes in reconstruction over predicted genes in genome against genome size for selected eukaryotic metabolic reconstructions. The three reconstructions with the highest ratio of genes in reconstruction per genes in genome are highlighted. bna572+ has a comparable ratio as *i*LB1025 and *i*LB1027_lipid, *i*TO977 has a higher ratio. Compared to *i*TO977 and bna572+, *i*LB1025 and *i*LB1027_lipid contain more reactions. The number of reactions in the respective reconstructions is used to scale the circle diameters. Note the discontinuous x-axis. Abbreviations: AraGEM: *Arabidopsis thaliana* [73]; bna572+: *Brassica napus* [74]; AlgaGEM: *Chlamydomonas reinhardtii* [75]; *i*RC1080: *Chlamydomonas reinhardtii* [39]; *i*RS1563: *Zea mays* [76]; *i*LB1025 and *i*LB1027_lipid: *Phaeodactylum tricornutum*, this study; *i*TO977: *Saccharomyces cerevisiae* Sc288 [77]; Recon2: *Homo sapiens* [78]; *i*MM1415: *Mus musculus* [79].

In order to assess *i*LB1027_lipid’s overall model coverage, we compared the ratio of genes accounted for in the reconstruction to genes predicted in the genome against the genome size for different eukaryotic organisms, namely *Arabidopsis thaliana*, *Brassica napus*, *Chlamydomonas reinhardtii*, *Zea mays*, *Saccharomyces cerevisiae*, *Homo sapiens* and *Mus musculus* (Fig 5). The considered reconstructions span a large range in genome size. The *i*LB1027_lipid model includes a higher ratio of genes in reconstruction per genes in genome (10%) than the median of all models (6%). *B*. *napus* (bna572+) has a comparable ratio of genes in the reconstruction (996) to predicted genes in the genome (9873) but contains far fewer reactions (671). The only model with a higher ratio belongs to the well-studied model organism *S*. *cerevisiae*, though this model *i*TO977 also contains fewer total reactions.

### Carbon partitioning

Recently, there has been a focus on using diatoms for biotechnological applications such as biofuel production, because of their high rate of neutral lipid accumulation [80,81]. Maximization of lipid biomass is a prerequisite for optimizing biofuel production in diatoms. Typical strategies for neutral lipid accumulation in *P*. *tricornutum* involve environmental stress, such as nitrogen or phosphorous limitation [37]. However, nutrient stress induced TAG accumulation also initiates growth arrest. TAGs store not only fixed carbon but also photosynthetically derived reducing equivalents. Storage of photosynthetically derived electrons into biomass also serves as photoprotection in diatoms [82].

**Fig 6:**
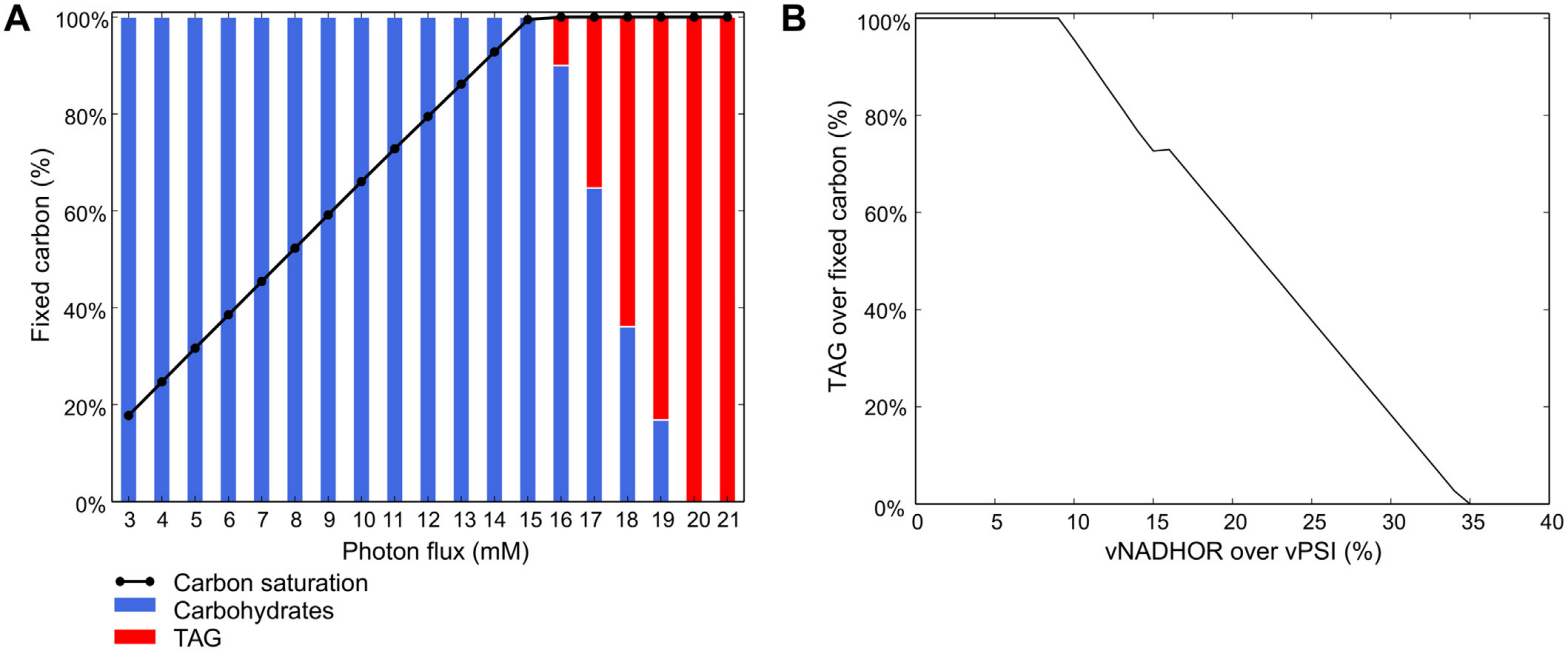
Light-dependent carbon partitioning. (A) Simulations indicated as photon uptake exceeds carbon uptake, excess redox potential is stored in triacylglycerol. The saturation of carbon uptake is shown in black. (B) Percent of carbon fixed in TAG against percent of metabolite flow through NADHOR (vNADHOR; EC 1.6.5.3,1.6.99.3) over metabolite flow through PSI (vPSI; EC 1.97.1.2) at a super-saturating photon uptake of 22 mM. According to our simulations TAG accumulation is inversely proportional to energetic coupling. TAG accumulation is prohibited when at least 35% of photosynthetically fixed electrons are redirected to the mitochondria.

Using the genome-scale model, we investigated the light-dependent partitioning of fixed carbon between storage carbohydrates and storage lipids, as shown in Fig 6A. Carbon fixation increased linearly with photon flux until saturation at the upper bound of CO_2_ uptake (experimentally determined, see Materials and Methods). Demand reactions added to the model allowed dynamic allocation of carbon and redox power into storage compounds and ensured mass balance with nutrient uptake. Resources could be fixed into biomass via nitrate reduction into ammonia, sulfate reduction into DMSP, carbohydrates or a representative TAG (see Materials and Methods). Prior to saturation at a photon uptake of 16 mM, all of the fixed carbon was stored as carbohydrates (see Fig 6A). Upon saturation, excess redox potential was stored as lipid and then as ammonia when all fixed carbon has been stored as TAG. No accumulation of DMSP was predicted.

### Energetic coupling between mitochondria and plastid

A recent, in depth characterization of photosynthetic electron flux in *P*. *tricornutum* enabled high quality constraints to be applied to the photosystem (Table 3). Results in Bailleul *et al*. indicated cyclic electron flow (CEF_h) accounted for approximately 30% of total electron flow at low irradiances and as low as 5% at high irradiances [83]. Fixing the CEF reaction boundaries to 0.3 mM approximated these ratios. Water-water reactions (plastid terminal oxidase (PTOX, EC 1.10.3.11), and Mehler reaction) constituted approximately 10% of the total electron flow. To allow the electron flow into these reactions to scale with photon uptake *in silico*, 5% of electron flow through the cytochrome b6f complex (CBFC_u) was routed to elemental oxygen mimicking the electron drain to PTOX while 5% of the electron flow through photosystem I (PSI_u) was committed to a Mehler-like reaction. Combined, these accounted for the 10% of electron flow to water-water reactions. Independent PTOX and Mehler reactions in the model are blocked by default but the boundaries can be adjusted to fit experimental results that deviate from the 10% value. In accordance with Bailleul *et al*.’s findings, the model predicts the use of mitochondrial oxidative phosphorylation to balance ATP and NADPH ratios.

**Table 3:** Photosynthetic electron flow constraints as determined by Bailleul *et al*. [83].

Abbreviation	Description	Constraint
CEF_h	Cyclic electron flow around PSI	LB = UB = 0.3 mM
FNOR_h	Ferredoxin:NADP^+^ Oxidoreductase	5% electron flow to Mehler reaction
CBFC_u	Cytochrome b6f complex	5% of electron flow to PTOX
PTOX_h	Plastid terminal oxidase	Default bounds set to 0 flux
MEHLER_h	Mehler reaction	Default bounds set to 0 flux
Constraint e-flow	Energetic coupling of mitochondria and plastid	νNADHOR—C ∙ νPSI ≥ 0

The model abbreviations refer to *in silico* reaction or metabolite identifiers. Abbreviations: LB, lower bound of reaction flux; UB, upper bound of reaction flux; νNADHOR, metabolite flow through the mitochondrial NADH:ubiquinone oxidoreductase; νPSI, metabolite flow through photosystem I, a proxy for total electron flow; C, a scalar value representing the percent of photosynthetically derived electrons coupled to mitochondrial respiration.

The model did not initially predict the use of the alternative oxidase (AOX, EC 1.10.3.11) to vent excess reducing equivalents. Our results predicted that flow of reductant from the plastid to the mitochondria was dependent on the ATP needs of the cell; however the results of Bailleul *et al*. suggest that this ratio is fixed over a range of low to moderate light intensities. To simulate the observed energetic coupling between the mitochondria and plastid, an inequality constraint was added to the model. This constraint forced a minimum amount of the photosystem flux to be routed to the mitochondrial electron transport chain. Upon adding energetic coupling, the model predicted AOX was a primary electron sink at high irradiances. Additionally, the energetic coupling affected accumulation of neutral lipid biomass. Storage of lipid biomass was inversely proportional to energetic coupling with TAG accumulation being abolished when at least 35% of photosynthetically fixed electrons were redirected to the mitochondria at super-saturating photon uptake (Fig 6B). Since lipid biosynthesis is dependent on plastid localized reducing power, it is possible that energetic coupling of the mitochondria and plastid is an inherent limit on the accumulation of neutral lipids, as predicted by the model. These results indicate that disrupting the energetic coupling of the plastid to the mitochondria while upregulating plastid lipid biogenesis and taking advantage of increased NADPH pools in AOX knockdown lines may result in increased TAG accumulation during exponential phase while alleviating the observed growth defect [83]. This would allow for the decoupling of growth process (e.g. nutrient limitation) from TAG production and increase overall yields of biofuel precursors.

**Fig 7:**
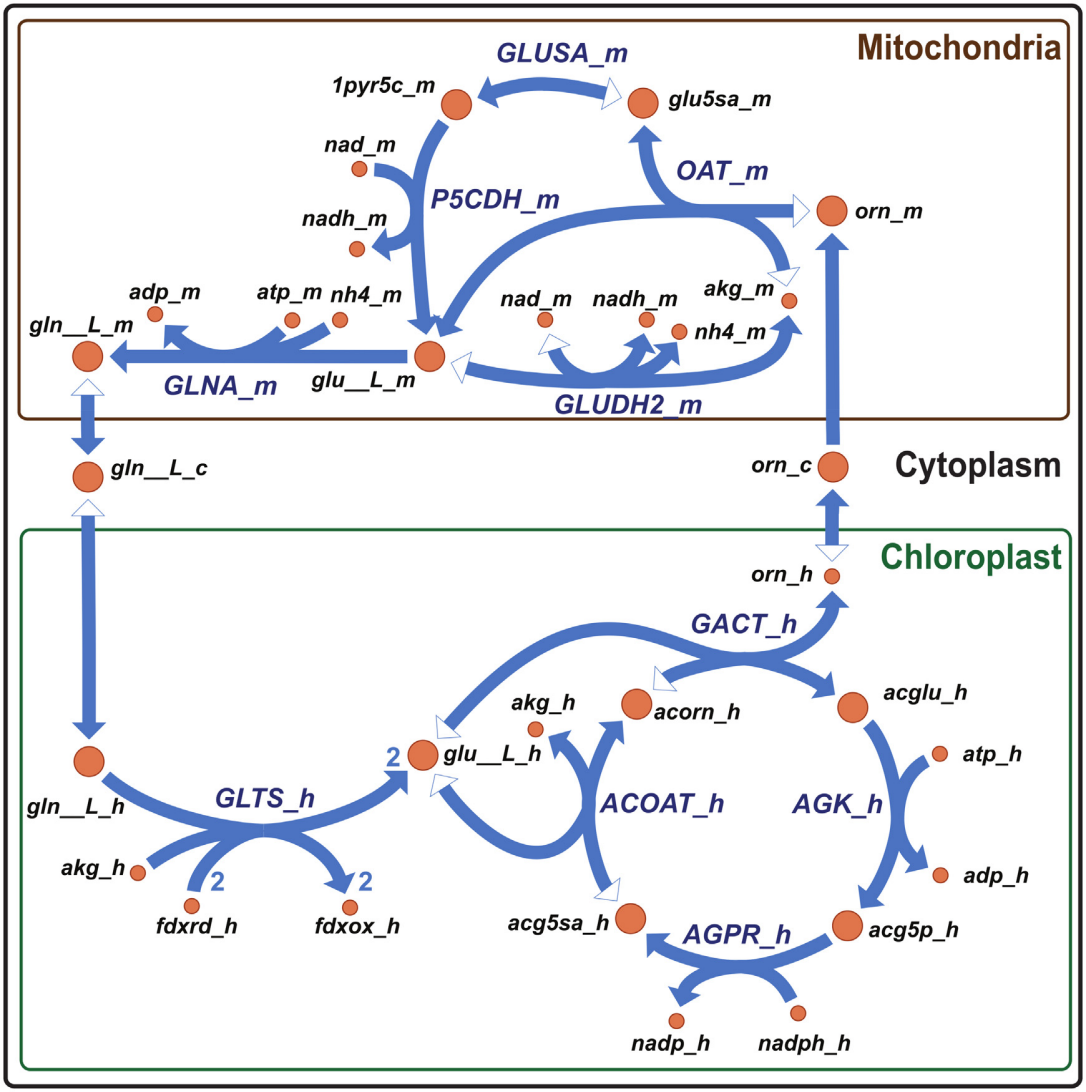
Chloroplastic ornithine cycle as revealed by the model. Metabolic network usage of a chloroplastic ornithine cycle is shown under a saturating photon constraint of 16 mM allowing maximum carbon uptake. Minor reactants and products are omitted for visual clarity (i.e., water, protons and phosphate). Metabolite and reaction abbreviation suffixes indicate cellular compartment; c, cytosol; h, chloroplast; m, mitochondria. Reversible reactions are indicated by arrowheads at both ends. The filled arrowhead indicates the direction in which the reaction is running, i.e. from substrate (open arrowhead) to product (filled arrowhead). Abbreviations used: ACOAT, acetylornithine transaminase; AGK, acetylglutamate kinase; AGPR, N-acetyl-δ-glutamyl-phosphate reductase; GACT, glutamate N-acetyltransferase; GLNA, glutamine synthase; GLTS, glutamate synthase (ferredoxin dependent); GLUDH2, glutamine dehydrogenase (NAD dependent); GLUSA, glutamate semialdehyde degradation (spontaneous); OAT, ornithine aminotransferase; P5CDH, 1-pyrroline-5-carboxylate dehydrogenase; acorn, N-acetylornithine; acglu, N-acetyl-L-glutamate; acg5p, N-acetyl-L-glutamate 5-phosphate; acg5sa, N-Acetyl-L-glutamate 5-semialdehyde; adp, ADP; akg, α-ketoglutarate; atp, ATP; fdxox, ferredoxin (oxidized); fdxrd, ferredoxin (reduced); gln__L, L-glutamine; glu__L, L-glutamate; glu5sa, L-glutamate 5-semialdehyde; nad, NAD^+^; nadh, NADH; nadp, NADP^+^; nadph, NADPH; nh4, ammonium ion; orn, ornithine; 1pyr5c, (S)-1-Pyrroline-5-carboxylate.

The mechanism by which reducing equivalents are shuttled to the mitochondria during energetic coupling is still unknown. In addition to the malate shuttle as proposed by Bailleul *et al*., our reconstruction uncovered a previously undescribed plastid ornithine biosynthetic pathway (Fig 7) that may represent an important metabolic connection between plastid and mitochondria. The compartmentalization pipeline indicated plastid targeting of acetylglutamate kinase (AGK_h, EC 2.7.2.8), N-acetyl-γ-glutamyl-phosphate reductase (AGPR_h, EC 1.2.1.38), acetylornithine transaminase (ACOAT_h, EC 2.6.1.11), and ornithine acetyltransferase (GACT_h, EC 2.3.1.35). Biomass yield simulations suggested that *in silico* the ornithine-glutamine shuttle is used to transfer reducing equivalents generated by photosynthesis to the mitochondria. Four photosynthetically derived electrons are used; two by the oxidation of ferredoxin molecules by plastid glutamate synthase (GLTS_h, EC 1.4.7.1) and two via oxidation of NADPH by AGPR_h. Ornithine is then proposed to be shuttled from the plastid to the mitochondria. The activity of 1-pyrroline-5-carboxylate dehydrogenase (P5CDH_m, EC 1.2.1.88) and glutamine dehydrogenase (GLUDH2_m, EC 1.4.1.2) produce NADH further suggesting that this novel ornithine-glutamate pathway coupling these two organelles is possible.

Storage of metabolites such as glutamine and ornithine could serve a photoprotective role by sequestering reducing equivalents as well as assimilated nitrogen. Indeed when intermediates of this ornithine shuttle were allowed to accumulate during simulations, the model predicted they were preferred over TAG biosynthesis. Ornithine concentrations were previously investigated in the context of the diatom ornithine-urea cycle (OUC) [5]. Although one of the most abundant metabolites in the cell, ornithine levels were not correlated with OUC intermediates, which indicated a possible alternative function [5]. We hypothesize storage of reducing power and electron transport into the mitochondria, potentially coupled to OUC consumption, is this alternative function.

## Conclusion

Our assembled reconstruction represents the current, comprehensive biochemical, genetic, and genomic knowledge about *P*. *tricornutum* and contains information such as reaction stoichiometry and associations between genes and reactions. We especially focused on lipid metabolism since diatoms are attractive candidates for industrial-scale lipid production [67,84]. The reconstruction is anticipated to facilitate model-driven exploration of the organism’s complex metabolism and hypothesis generation. Furthermore, the manually curated metabolic network facilitates visualization and analysis of different data types including metabolomics, fluxomics or common genomic data such as RNA-Seq. We have demonstrated that the model reflects the known biochemical composition of these algae in defined culture conditions (Fig 4) and that it enables the study of light-dependent carbon partitioning (Fig 6). Diatoms thrive in highly dynamic environments and this model will provide a template for future studies that aim to understand how diatoms balance photosynthesis and heterotrophic metabolism over light-dark cycles or the stochastic supply of nutrients. This model will also enable metabolic engineering strategies to improve the use of *P*. *tricornutum* for biotechnological applications.

## Supporting Information

S1 File Supplementary methods and figures. (DOCX)

S2 File Supplementary tables A-O. (XLSX)

S3 File Genome-scale metabolic model of *P*. *tricornutum* in MAT and SBML format. (ZIP)

S4 File MATLAB scripts used for model simulation. (ZIP)
