# Supplementary material for: Genome-Scale Model Reveals Metabolic Basis of Biomass Partitioning in a Model Diatom
Source: PLoS One. 2016 May 6;11(5):e0155038. doi: 10.1371/journal.pone.0155038 (PMC4859558; doi:10.1371/journal.pone.0155038)
Supplement: S1 File — (DOCX) [file pone.0155038.s001.docx]

# S1 File

# Genome-scale model reveals metabolic basis of biomass partitioning in a model diatom

Jennifer Levering, Jared Broddrick, Christopher L Dupont, Graham Peers, Karen Beeri, Joshua Mayers, Alessandra A Gallina, Andrew E Allen, Bernhard O Palsson, Karsten Zengler

# Section A: Detailed description of the subcellular localization prediction pipeline

In step one of our pipeline (Fig A) all nuclear-targeted proteins were identified using predictNLS. If the protein had no nuclear targeting signal, TargetP and SignalP 4.0 were used to predict the general protein localization. If the protein without the signal peptide had a chloroplast transit peptide (cTP) score lower than 0.5, the presence of an endoplasmic reticulum (ER) retention signal was tested. If none was detected and the +1 position of the cleavage site was F, W, Y, or L or the cTP score was greater than or equal to 0.5, the protein was considered to be chloroplast targeted; otherwise ER targeted. If no ER retention signal was found and TargetP predicted another localization or the protein was not a signal peptide, mitochondrial targeting was checked using HECTAR, SignalP 3.0 and Mitoprot. If the protein also had a peroxisomal targeting signal it was predicted to be localized in both the mitochondria and the peroxisome. If the protein was not predicted to be mitochondrial targeted, it was screened for the presence of a peroxisomal targeting signal and if this was absent the presence of transmembrane helices was checked using TMHMM. If no transmembrane helix was present, the protein was categorized as cytoplasmic, otherwise it might be localized in the ER.

# Section B: Development of FTIR models of *Phaeodactylum tricornutum* biomass

## *P. tricornutum* FTIR calibration

A twenty liter culture of *P. tricornutum* was grown with rapid bubbling and stirring to prevent CO_2_ limitation with 2f-N-limit-Si media, essentially f/2 media with the traditional phosphate, and trace metal mix concentrations multiplied by four, silicate eliminated, and nitrate concentrations only doubled. Cells were grown in 100 µmol photon m^-2^ s^-1^ light with a diel cycle of 14 hours light and 10 hours dark. *In vivo* chlorophyll a fluorescence was monitored twice daily using a fluorometer (Turner Instruments, Inc.) This allows for high cell densities to be attained but also an attendant self-shading with biomass accumulation and eventual nitrogen starvation. This type of culture experiences a broad and smooth transition between ranges of biochemical compositions. Cell pellets were taken from culture twice daily by centrifugation (7000 ***g*** for 10 minutes), at the end of the dark period and middle of the light period, and centrifuged at 9000 ***g*** with transfer. After lyophilization and grinding to a fine powder, this material was used for biochemical measurement of proteins, carbohydrates, lipids and fatty acid methyl ester (FAME). In addition, all samples were scanned using Fourier transform infrared (FTIR) spectroscopy and the biochemical measurements were used in a calibration against their corresponding FTIR peaks, with the methods described below. These dual measurements were then used to develop linear models with spectra peak height and *P. tricornutum* biochemical composition (essentially linear correlation curves) as done by Mayers *et al.* [1].

A typical *P. tricornutum* FTIR profile is shown in Fig 1. Total carbohydrates were extracted from 20 samples in triplicates, and % carbohydrates per dry weight values were used to calibrate FTIR peak corresponding to C-O-C at 1045-1025 cm^-1^, representing polysaccharides and siloxane (Table A, S2A Fig). Total proteins were extracted from 19 samples in triplicates, and the resulting % protein per dry weight values were used to calibrate FTIR peak corresponding to amide bond II at 1510-1500 cm^-1^ (Table A, Fig B). As our cultures were grown without silica, we can disregard the siloxane input. Total lipids were extracted from 14 samples in triplicates, and % lipids per dry weight values were used to calibrate FTIR peak corresponding esters of lipids and fatty acids at 1750-1725 cm^-1^ (Table A, Fig C). The difference in the number of extractions used in FTIR calibrations are due to intermittent failures in the traditional biochemical techniques and lack of biomass, indeed, it is this failure rate, along with biomass demands, use of toxic chemicals, and general tedium that inspired the development of FTIR models. Notably, the approach taken does not require replications of individual time points, rather a gradient in the biochemical composition, as the models depend upon correlation, rather than means testing. As both variables that are compared are independently and directly inferred, the correlations are entirely empirical. In the case of proteins, carbohydrates, and lipids, we were able to achieve statistically relevant and high correlations between FTIR peak heights and chemically determined biochemical composition. These correlations were then used to determine biochemical composition for all time points across the growth curve. All correlations were done using model 2 linear regressions in R using Calc_lm.r statistical tests.

## Sample processing

Cells were harvested by centrifugation for 10 min at 4°C under 10,000 ***g***, the pellet was washed once with equal volume of fresh 0.5 M NH_4_CO_3_ to remove salts, washed pellets were transferred to 50 ml tube, washed again and flash frozen in dry ice. Frozen pellets were freeze dried under vacuum using a benchtop manifold freeze dryer (Millrock) for 12 h. Dry pellets were ground and freeze dried for an additional 30 min to remove moisture absorbed while processing.

## FTIR

The IRPrestige-21 (Shimadzu) with Single reflection ATR (PIKE Technologies) was used for the FTIR scans and the generation of calibration curves. Each sample was scanned a minimum of three times, with 4 mg dry biomass was used for each scan, and scan averaging was done using the IResolution software.


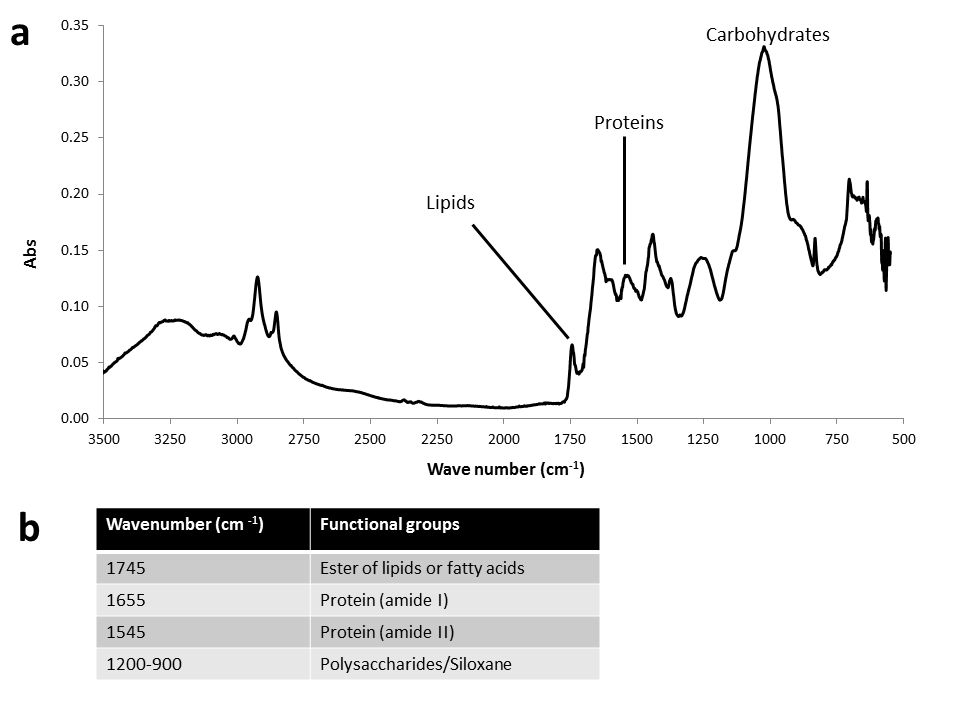


**Table A. FTIR wavelengths used for the calibration.**

## Protein quantification

Two to four mg dry weight was placed in 1.7 ml tubes and resuspended in 300 μl of a buffer containing 50 mM Tris-HCl pH 6.8, 200 mM NaCl and 1 mM PMSF. The samples were sonicated after which 100 μl of sample buffer containing 100 mM Tris-HCl pH 6.8, 6.5% glycerol, 1.6% SDS, 12.5% β-mercaptoethanol and 0.005% Bromophenol Blue was added. The samples were then boiled for 15 min and taken for the analysis; 5 μl of the samples were spotted in duplicates on Whatman paper that was then stained with Coomassie for 30 min and washed with destain. Paper was dried on a heat block after which the paper containing the protein samples was cut, placed in a multiwall plate and incubated with 0.5 ml 3% SDS for 1 h at 37^o^C in a shaker. 200 μl from each plate was transferred to a 96 well plate and read at 595 nm. A calibration curve was obtained using 1 mg ml^-1^ BSA in sample buffer in the range of 1-20 μg [2].

## Calorimetric assessment of carbohydrate content

4 mg of dry weight was boiled in 2 ml 1M H_2_SO_4_ for 60 min followed by centrifugation at 5500 ***g*** for 10 min. 100 μl of the supernatant was taken into a boiling tube to which 2.5 ml of undiluted H_2_SO_4_ was added following with 0.5 ml phenol. Following 20 min incubation, 200 μl of each sample was transferred to a 96 well plate and read at 485 nm. The carbohydrate content was calculated using the equation from a standard curve made with glucose [3,4].

## Lipid extraction

Total lipids were extracted according to Bligh and Dyer [5]. 10-12 mg dry weight was measured and placed in Teflon tube to which 4 ml chloroform and 2 ml of methanol were added. Samples were mixed and placed in ice water sonicator for 60 min and then placed at 4°C in the dark overnight. 1 ml of 0.09% (w/v) NaCl was added following centrifugation at 2000 ***g*** for 10 min at 4°C. The bottom phase was recovered into pre-weighted glass vials. Lipids were dried under nitrogen after which the vials were weighted again; the vial weight was subtracted from the total to obtain the mass of the lipids.

## Fatty Acid Methyl Ester (FAME) analysis

0.5 ml of 0.5 M KOH in MeOH, 0.5 ml Ottawa sand, and 80 µL of an I.S. mix containing C11:0 FFA/C13:0 TAG/C23:0 FAME were added to dried samples in 4 ml glass vials. The samples were capped with PTFE-lined caps and placed in a GenoGrinder at 1200 ***g*** for 10 min. Subsequently, the samples were heated at 80°C for 30 min then placed in the GenoGrinder again at 1200 ***g*** for 5 min. Next, the samples were uncapped and 0.5 ml of 14% BF3 in MeOH was added. The samples were recapped and placed in the 80°C-heating block again for 30 min. After heating the samples were placed in the GenoGrinder for 5 min at 1200 ***g***. The samples were then uncapped and 2 ml of n-heptane followed by 0.5 ml of saturated NaCl were added. Last, the samples were placed in the GenoGrinder a final time at 1200 ***g*** for 1.5 min, centrifuged at 1000 ***g*** for 3 min, and the upper phase was sampled directly for GC-FID analysis.

## Total organic carbon (TOC) and total nitrogen (TN) analysis

TOC/TN was analyzed using TOC-L and TNM-L (Shimadzu). 20 ml of culture were collected and diluted 1:1 in milliQ water. The sample was then divided in two. 20 ml were analyzed as is and the remaining 20 ml were passed through a 0.2 mm glass filter (Pall) which retained cells. These represented the total carbon and nitrogen in the entire sample and that contained in solution, respectively. Both were acidified to pH 4.5 using 10% HCl (1/1000th of the total volume) to degas DIC. Particulate TOC/TN was calculated by subtracting the dissolved TOC/TN from total.

# Section C: Biomass objective function calculation

The biomass objective function accounts for all known biomass components and their fractional contributions to the overall cellular biomass. To assemble this reaction, the cellular content of each component (DNA, RNA, protein, pigments, carbohydrates, membrane lipids and TAGs) in % (wt/wt) and the fractional contributions in mmol gDW^-1^ is needed. Each biomass metabolite is normalized to 1 gDW using correction factors specified in Tables D-L in S2 File.

## DNA

The nucleotide sequence for the nuclear, mitochondrial and plastid genomes were analyzed for their A, T, G and C content. The genome was doubled to account for both strands and the nuclear genome was doubled again to account for the diploid nature of the organism. Unidentified nucleotides in the nuclear genome (indicated by N) were distributed across all dNTPs in their respective ratios. The total value given is after accounting for strandedness and diploidy (see Table D in S2 File).

The total number of each nucleotide was converted into g per unit cell using the molecular weight of the respective deoxynucleotide monophosphate (these are the incorporated form) and Avogadro’s constant (N_A_ = 6.022×10^23^ mol^−1^) (see Table E in S2 File).

The relative abundance of each nucleotide was calculated based on the total nucleotides and reflects the molar ratio used for the stoichiometry in the biomass_DNA_c reaction; which was then normalized to 1 gDW DNA using the molar mass of the corresponding dNTP minus the mass of the pyrophosphate on the product side (see Table E in S2 File).

## RNA

The relative abundance of each nucleotide was the same as in DNA. The amount of RNA per cell was calculated using an RNA to DNA ratio of 8:1 based on the previous results [6]. Normalization of the stoichiometry to 1 gDW RNA in the biomass_RNA_c reaction accounted for the mass difference between dNMPs and NMPs (see Table E in S2 File).

## Protein

Values were taken from previous work [7] and reported as weight percent. Hydroxyproline, ornithine and GABA were not included in the biomass equation. The values for aspartate and glutamate were evenly split with asparagine and glutamine respectively due to base catalyzed deamination. The sum of the compounds weight percent was normalized to 100%. tRNA metabolites were used from the model in order to account for the energy required for charging the tRNA with an amino acid. Weight percent was converted to molar ratios by dividing by the molar mass and the stoichiometry was normalized to generate the biomass_pro_c metabolite. For the normalization, one ATP and two GTP (besides all other involved reactants) were added to account for the polymerase energy requirements (see Table F in S2 File).

## Pigments

Reported values for pigments [8] were averaged for all nitrogen sources during log phase resulting in 1.4% chlorophyll a gDW^-1^. Dividing by the molar mass resulted in mol chlorophyll a gDW^-1^; this molar value was used for subsequent ratios. Chlorophyll a to chlorophyll c and chlorophyll c1 to c2 ratios were taken from the thylakoid fraction [9]. Fucoxanthin to chlorophyll a, diadinoxanthin to chlorophyll a and β-carotene to chlorophyll a ratios were taken from literature [10]. The molar percent gDW^-1^ of pigments was multiplied by the molar mass and summed up resulting in percent pigments gDW^-1^ as shown in Table G in S2 File. The molar ratios were normalized to generate the stoichiometry for the biomass_pigm_h reaction. The total pigment amount was set as the initial value and was adjusted up or down based on the experimental Fv/Fm values for the culture.

## Carbohydrates

Carbohydrate distributions were based on published results [11,12]. The relative amount of each extracted fraction was interpolated from the results for late-log phase (days 10-14) [12]. The composition of the fractions was based on the log phase molar ratios of major sugars [11] normalized to 100%. Ribose was not included in the normalized results since the intracellular origin was not clear. Glucuronate and sulfate fractions were taken from Abdullahi *et al.* [12]. The percent sulfate was removed from the mannose pool to account for the sulfated mannose polysaccharide. The biomass equation precursors were the nucleotide diphosphate (NDP)-sugar moieties and the reaction biomass_carb_c was generated by modeling polymerization to the polysaccharide and release of the NDP. For more details on the carbohydrate fraction see Table H in S2 File.

## Lipids

The ratios of major lipid classes during nitrogen replete and nitrogen starved growth were based on previous work [13] (see Table I in S2 File). Lipids and TAGs are the only biomass components with dynamic stoichiometric coefficients, i.e. the coefficients change with every sample due to the variation in membrane lipid to TAG ratio. Exemplary, the stoichiometric coefficients for sample 9 are given in Table I in S2 File.

The stoichiometric coefficients for the metabolites that make up the lipid biomass components were determined using a linear programing (LP) strategy that satisfied the molar ratios of the lipid classes and experimentally derived FAME data. Briefly, a lipid stoichiometry matrix (**A**) was generated with all lipid metabolites in the model as the columns and the fatty acid and lipid head group building blocks as the rows. The equation **A**∙**x** = **b** was solved for **x**, where **b** was equal to the molar ratios of the fatty acids in the FAME data and the molar ratios of the lipid classes from Abida *et al.* [13]. To account for experimental error, a 10% deviation from the experimental values was allowed. The resulting vector **x** was equal to the stoichiometric coefficients for the lipid metabolites that satisfied the experimental data. These metabolites were used to generate the biomass_mem_lipids_c and biomass_TAG_c reactions and are given for sample 9 (day 5) in Table I in S2 File.

## TAG

The ratio of TAGs to membrane lipids during nitrogen replete and nitrogen starved conditions was taken from Abida *et al.* [13]. The rate of TAG increase was interpolated from Nile Red fluorescence curves normalized to the onset of stationary phase [14]. TAGs are 2% of total lipids during nitrogen repleted conditions and 66% of total lipids under nitrogen limitation. The metabolites used to construct the biomass_TAG_c reaction were selected via the LP strategy outlined above. For our culture at day 5 (sample 9) TAGs were estimated to be 22% of total lipids.

## Biomass component ratios

Percent dry weight values for lipids, proteins and carbohydrates were taken from the FTIR data. Percent dry weight values for pigments, DNA, and RNA were added to the totals. Lipids were split into TAGs and membrane lipids based on the interpolated values mentioned above. The sum of the total biomass was usually less than 100%. The remaining difference was split evenly between proteins and carbohydrates since FTIR tends to overestimate lipids. An example for sample 9 (day 5) harvested towards the end of exponential phase is shown in Tables J and K in S2 File; Table L in S2 File summarizes the biomass component ratios for all samples.

# Section D: Supplementary Figure


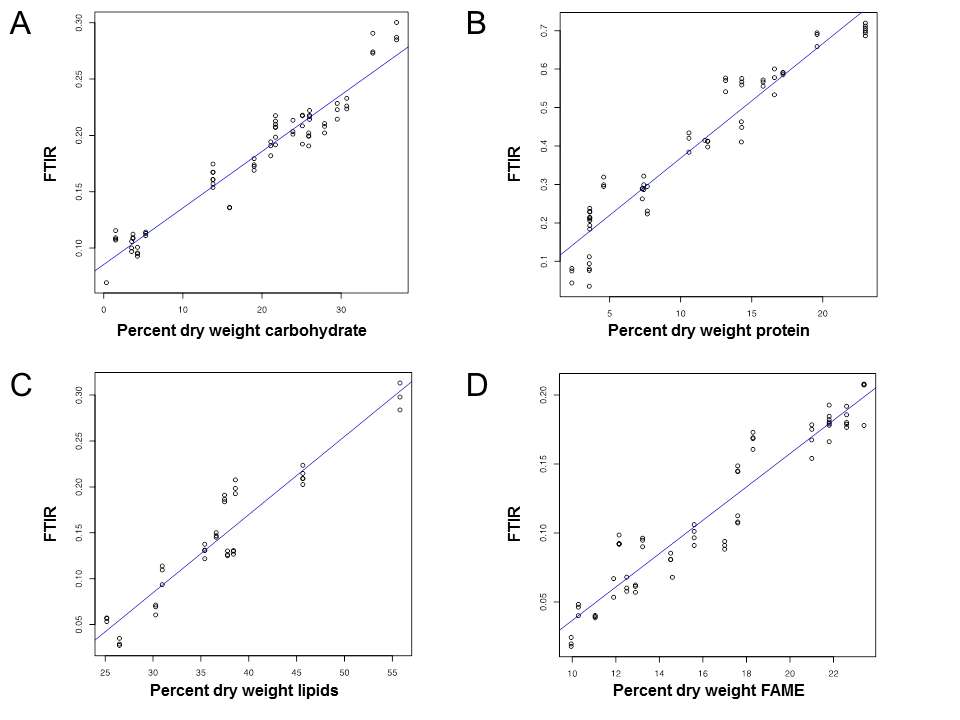


**Fig A. Calibration graphs.** (A) Calc_lm.r linear regression modeling of the percent dry weight protein measurements with the absorbance at the protein peak in an FTIR spectrum. The p-value for the curve fitting is 2.6x10^-35^ and the adjusted R^2^ value is 0.918. (B) Calc_lm.r linear regression modeling of the percent dry weight carbohydrate measurements with the absorbance at the carbohydrate peak in an FTIR spectrum. The p-value for the curve fitting is 5.2x10^-34^ and the adjusted R^2^ value is 0.935. (C) Calc_lm.r linear regression modeling of the percent dry weight lipid measurements with the absorbance at the lipid peak in an FTIR spectrum. The p-value for the curve fitting is 2.9x10^-20^ and the adjusted R^2^ value is 0.899. (D) Calc_lm.r linear regression modeling of the percent dry weight fatty acid methyl ester (FAME) measurements with the absorbance at the lipid peak in an FTIR spectrum. The p-value for the curve fitting is 1.1x10^-34^ and the adjusted R^2^ value is 0.909. For each biochemical measurement, multiple FTIR scans were performed.


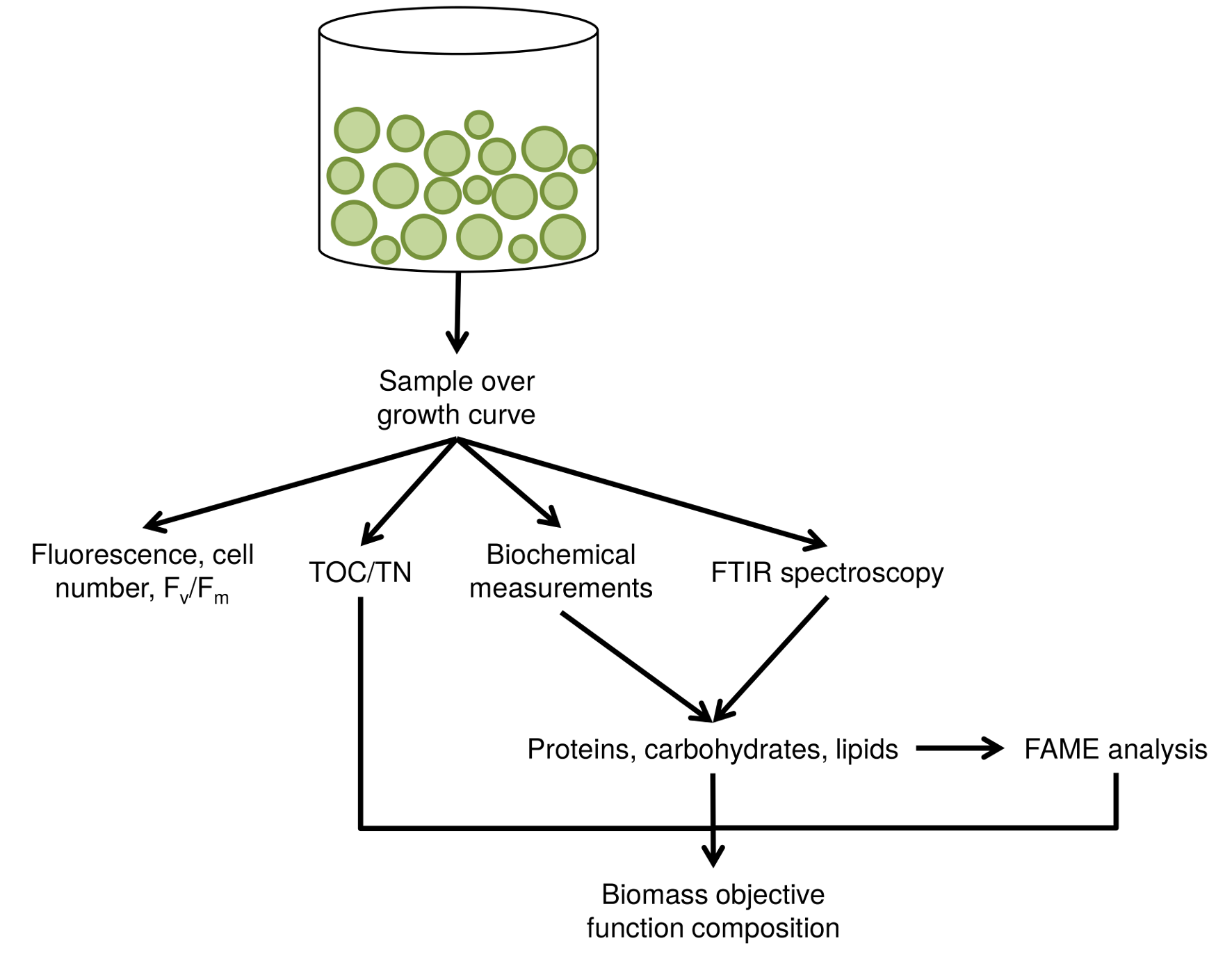


**Fig B. Summary of performed measurements and experimental workflow.** Samples were taken over a growth curve from nitrogen replete during exponential growth phase to nitrogen starved during stationary phase and fluorescence, cell number, F_v_/F_m_, total carbon (TOC), and total nitrogen (TN) were measured. Additionally, biomass composition in terms of proteins, carbohydrates and lipids was determined using traditional biochemical measurements which were correlated to FTIR spectra peak heights to develop models enabling high-throughput determinations of cellular biomass composition. For more detail on the experimental procedures see Section S2 and Materials and Methods.

# References

1. Mayers JJ, Flynn KJ, Shields RJ. Rapid determination of bulk microalgal biochemical composition by Fourier-Transform Infrared spectroscopy. Bioresour Technol. 2013;148: 215–220. doi:10.1016/j.biortech.2013.08.133

2. Ghosh S, Gepstein S, Heikkila JJ, Dumbroff EB. Use of a scanning densitometer or an ELISA plate reader for measurement of nanogram amounts of protein in crude extracts from biological tissues. Anal Biochem. 1988;169: 227–233. doi:10.1016/0003-2697(88)90278-3

3. DuBois M, Gilles KA, Hamilton JK, Rebers PA, Smith F. Colorimetric method for determination of sugars and related substances. Anal Chem. 1956;28: 350–356. doi:10.1021/ac60111a017

4. Templeton DW, Quinn M, Van Wychen S, Hyman D, Laurens LML. Separation and quantification of microalgal carbohydrates. J Chromatogr A. 2012;1270: 225–234. doi:10.1016/j.chroma.2012.10.034

5. Bligh EG, Dyer WJ. A rapid method of total lipid extraction and purification. Can J Biochem Physiol. 1959;37: 911–917.

6. Lourenço SO, Barbarino E, Lavín PL, Lanfer Marquez UM, Aidar E. Distribution of intracellular nitrogen in marine microalgae: calculation of new nitrogen-to-protein conversion factors. J Phycol. 1998;34: 798–811. doi:10.1080/0967026032000157156

7. Brown MR. The amino-acid and sugar composition of 16 species of microalgae used in mariculture. J Exp Mar Bio Ecol. 1991;145: 79–99.

8. Fidalgo JP, Cid A, Abalde J, Herrero C. Culture of the marine diatom *Phaeodactylum tricornutum* with different nitrogen sources: growth, nutrient conversion and biochemical composition. Cah Biol Mar. 1995;36: 165–173.

9. Owens TG, Wold ER. Light-harvesting function in the diatom *Phaeodactylum tricornutum*: II. Distribution of excitation energy between the photosystems. Plant Physiol. 1986;80: 732–738. doi:10.1104/pp.80.3.739

10. Veith T, Büchel C. The monomeric photosystem I-complex of the diatom *Phaeodactylum tricornutum* binds specific fucoxanthin chlorophyll proteins (FCPs) as light-harvesting complexes. Biochim Biophys Acta. 2007;1767: 1428–1435. doi:10.1016/j.bbabio.2007.09.004

11. Willis A, Chiovitti A, Dugdale TM, Wetherbee R. Characterization of the extracellular matrix of *Phaeodactylum tricornutum* (Bacillariophyceae): structure, composition, and adhesive characteristics. J Phycol. 2013;49: 937–949. doi:10.1111/jpy.12103

12. Abdullahi AS, Underwood GJC, Gretz MR. Extracellular matrix assembly in diatoms (Bacillariophyceae). V. Environmental effects on polysaccharide synthesis in the model diatom, *Phaeodactylum tricornutum*. J Phycol. 2006;42: 363–378. doi:10.1111/j.1529-8817.2006.00193.x

13. Abida H, Dolch L-J, Meï C, Villanova V, Conte M, Block MA, et al. Membrane glycerolipid remodeling triggered by nitrogen and phosphorus starvation in *Phaeodactylum tricornutum*. Plant Physiol. 2015;167: 118–136. doi:10.1104/pp.114.252395

14. Mus F, Toussaint J-P, Cooksey KE, Fields MW, Gerlach R, Peyton BM, et al. Physiological and molecular analysis of carbon source supplementation and pH stress-induced lipid accumulation in the marine diatom *Phaeodactylum tricornutum*. Appl Microbiol Biotechnol. 2013;97: 3625–3642. doi:10.1007/s00253-013-4747-7
